# Evaluation of the Morphological, Physiological and Biochemical Effects Induced by Coragen 20 SC in Some Non-Target Species

**DOI:** 10.3390/toxics11070618

**Published:** 2023-07-17

**Authors:** Cristina Maria Ponepal, Liliana Cristina Soare, Oana-Alexandra Drăghiceanu, Cristina Florina Mihăescu, Nicoleta Anca Șuțan, Monica Marilena Țânțu, Alina Păunescu

**Affiliations:** Natural Science Department, Faculty of Sciences, Physical Education and Informatics, University of Pitesti, 110040 Pitesti, Romania; cristina.ponepal@upit.ro (C.M.P.); cristina.mihaescu@upit.ro (C.F.M.); anca.sutan@upit.ro (N.A.Ș.); monica.tantu@upit.ro (M.M.Ț.); alina.paunescu@upit.ro (A.P.)

**Keywords:** Coragen 20 SC, chlorantraniliprole, non-target species, *Perca fluviatilis*, *Triticum aestivum*

## Abstract

Coragen 20 SC is an insecticide based on chlorantraniliprole that is applied on many crops. Considered an effective product with an incremental cost-benefit ratio, it has been widely used globally. Residual pesticides affect non-target organisms, so it is necessary to explore the possible effects induced by these xenobiotics on different species. This work aimed to assess some morphological, physiological and biochemical effects induced by Coragen 20 SC on two non-target species: *Perca fluviatilis* (Linné, 1758) and *Triticum aestivum* L. The concentrations used were the same for all tested species (0.0125, 0.025 and 0.05 mL L^−1^), and the experiments were of the acute, subchronic and chronic type. The toxicological effects of Coragen 20 SC on perch recorded behavioral changes, a decrease in respiratory rate and oxygen consumption, an increase in blood glucose levels and a decrease in the number of erythrocytes and leukocytes. The results obtained from the evaluation of Coragen 20 SC toxicity using the *Triticum* test indicate a weak to moderate phytotoxicity for the considered parameters at the applied doses. Only the assimilatory pigments were significantly modified at the concentration of 0.025 mL L^−1^ for the growth of the axial organs and the wet and dry weight, with the changes obtained not being statistically significant.

## 1. Introduction

Pesticides derived from chemical syntheses are the best examples of compounds whose use is risky because they spread all over the environment. Although they are produced in a few countries, their use has become global due to exportation. Water ecosystems are highly exposed to pesticides, with different sources of contamination and action level. While the use of pesticides has many beneficial effects, such as increasing productivity by reducing losses due to various pests, aquatic and terrestrial ecosystems are increasingly affected by these compounds. Through bioconcentration and biomagnification, these compounds are transmitted along food chains, affecting various non target organisms and even human populations. 

The use of pesticides in agriculture has resulted in several consequences, including the decline of fish populations [1] and fish biodiversity [2]. Fish can be exposed to pesticides either through their gills or from contaminated food. Given the significant negative impact of pesticides on the environment, particularly organochlorine and organophosphorus compounds, there is a growing need for new, more environmentally friendly, and selective product. One such product is chlorantraniliprole (CAP), which has been developed worldwide by DuPont (Tokyo, Japan) and is marketed under the trade name Rynaxypyr^®^ [3]. CAP was first registered in the Philippines in 2007 [4] and has since been used in various agricultural regions globally [5], showing promising results [6]. However, there are limited studies on the toxicological risks associated with this insecticide [3]. CAP belongs to the anthranilic diamide class and was first introduced in 2008 [7]. It is now used on a global scale [8]. 

Anthranilic diamides constitute a recently emerged group of insecticides with a highly specific and distinctive mode of action compared to other insecticide groups. They act as activators of ryanodine receptors (RyR), which are intracellular non-voltage calcium channels present in the sarcoplasmic reticulum (SR) of muscles and the endoplasmic reticulum (ER) of non-muscle cells [3]. Their selectivity is attributed to their reduced toxicity to mammals, which is 300 times lower than their toxicity to insects [9]. Fish possess only two types of ryanodine receptors (RyRA and RyRB) [10]. While the risk of CAP insecticide appears to be low for fish, the selectivity of its toxicity among different fish species remain unclear [11]. Direct spray, spray drift, leaching to ground water, atmospheric deposition, and runoff represent potential transport mechanisms of CAP to aquatic and terrestrial organisms [12]. Excessive use of CAP may result in persistent contamination of treated foods and adverse effects on human well-being [13]. 

The insecticide exhibits high persistence in soils and aquatic sediments [14]. Degradation of CAP in water primarily occurs through hydrolysis, or it is catalyzed by acids or bases that come into contact with the compound [15]. CAP is stable at a pH of 4 and 7 [16], DT50 being 10 days at pH 9 [17]. Photolysis is an important degradation pathway for CAP compared to its degradation/dissipation in water [16]. The bioconcentration capacity of the CAP insecticide is moderate (BCF: 13–15), and the product is rapidly eliminated (CT50: 1.5 days, CT90: 8.9 days). In fish, the main metabolite is IN-ECD73, of which 95% is eliminated within two weeks of exposure [16]. In a study on the bioaccumulation capacity of the CAP insecticide carried out on fish belonging to the species *Lepomis macrochirus* that were exposed for 14 days to concentrations of 0.0132 and 0.138 mg insecticide L^−1^ exposure followed by a purification period of 21 days, a maximum level was found after 11 days of exposure, and the purification capacity (CT50 and CT90) for whole fish were 1.5 and 8.9 days, respectively [14]. CAP degrades rapidly in fish to IN-ECD73 and polar metabolites [14]. 

Coragen 20 SC is an insecticide based on CAP applied for use in many crops to target Lepidoptera and some Coleoptera, Diptera and Isoptera species [18]. It exhibits high biological activity [19]. Recently considered as an effective insecticide with an incremental cost–benefit ratio [20], Coragen has widespread global use. The water solubility of Coragen 20 SC at 20 °C is 0.880 mg L^−1^ at pH 7 and 0.971 mg L^−1^ at pH 9 [14]. Fish are commonly utilized as experimental models for assessing toxicity in aquatic ecosystems [21]. 

The choice of perch as a bioindicator species for CAP insecticide pollution is based on its wide distribution in rivers in Romania and the facilities for its capture and maintenance under laboratory conditions. At a single, short-term exposure, the insecticide has low toxicity to fish [14]; at long-term exposures, however, the toxicity of the product increases [7,22,23] 96 h LC50: *Ictalurus punctatus* > 13.4 mg L^−1^; *Lepomis macrochirus* > 15.1 mg L^−1^; *Oncorhynchus mykiss* > 13.8 mg L^−1^. The NOEC values reported in chronic tests are 0.11–1.28 mg L^−1^ [16]: NOEC 32 days for *Cyprinodon variegatus* at 1.28 mg L^−1^, and NOEC 28 days for *Oncorhynchus mykiss* at 0.110 mg L^−1^ [24]. No changes in hatchability, survival, weight gain and length were found in trout fingerlings exposed to CAP for 90 days in continuous flow tests [14]. However, significant larval abnormalities were found, resulting in an NOEC value of 0.110 mg CAP L^−1^ [14]. 

Tests on aquatic plants and algae indicate low to moderate toxicity of CAP. Therefore, for duckweed, the 14-day EC50 is >2000 µg CAP L^−1^, and for algae, the 120 h EC50 is >2000 -> 15,100 µg CAP L^−1^ [16]. The same document indicates the possibility of accumulation through repeated administration and persistence in soil because the half-life is one year. Residual pesticides affect non-target organisms, so the possible effects induced by these xenobiotics on different species need to be explored. USEPA developed the Test Guidelines for Pesticides and Toxic Substances, which are generally intended to meet the toxicity testing requirements for terrestrial and aquatic animals and plants. Because plants are an important part of each ecosystem, many tests target primary producers. Agricultural species are generally used for toxicity assessment tests in terrestrial ecosystems [25]. 

For terrestrial plants, the standard tests establish vegetative vigor, seedling emergence and seedling growth, early seedling growth toxicity, plant uptake, and translocation. Growth inhibition and biomass accumulation are recommended parameters for testing the toxicity of chemicals in plants. Xenobiotic agents also induce changes in the chlorophyll content [26]. Chlorophyll is an important compound because it is responsible for photosynthesis and is an indicator of plant health [27]. Pesticides may reduce the chlorophyll pigments, photosynthetic efficiency, and protein content [28], and they may influence the absorption of vital mineral nutrients by the vascular plants [29]. 

The *Triticum* test is widely used for testing therapeutic products and other xenobiotics [30], with this species also being recommended in standard tests [31]. The widespread use of pesticides to protect crops may not only result in pesticide residues within the plant, but can also affect the metabolic processes during growth, causing abnormal development [32]. 

This work aimed to assess the toxicological effects induced by Coragen 20 SC on two non-target species: perch—*Perca fluviatilis* (Linné, 1758) and weat—*Triticum aestivum* L. by evaluating oxygen consumption, respiratory rate, blood figure elements, glucose and behavioral change for the former, and growth parameters (root and stem length, wet and dry weight) and physiological changes (chlorophyll *a*, *b,* and carotenoids content) for the latter. 

## 2. Materials and Methods

### 2.1. The Evaluation of Coragen-Induced Toxicity on Fish

The experiments were carried out on males and females of perch—*Perca fluviatilis* (Linné, 1758) with an average weight of 18.42 ± 2.14 g, caught from the Argeș River. The fish were acclimatized for 10 days to laboratory conditions, during which they were fed ad libitum once a day, after which they were divided into four groups of 10 specimens each, as follows: the control group that was without the addition of insecticides, and three experimental groups exposed to Coragen 20 SC. The insecticide was diluted in double-distilled water at pH 7. The 0.01, 0.025 and 0.05 mL L^−1^ Coragen formulations contained 2.5, 5 and 10 mg L^−1^ CAP, respectively. The tested concentrations were established on the basis of specialist work and preliminary tests (survival in the case of fish). Data from the specialized literature indicate the low toxicity of CAP in fish (>10–100 mg L^−1^), which is why we chose the maximum working concentration of 10 mg/L (0.05 mL·L^−1^ Coragen 20 SC); the test was carried out for 14 days. 

The test method used was semi-static, with the solution being refreshed every 24 h. The fish were contained with continuous aeration in 100 mL aquariums, with continuous aeration of water (dissolved oxygen 7.53 ± 0.32 mg L^−1^, pH 7.83 ± 0.65, total hardness 100 mg L^−1^ CaCO_3_) and with a natural light/dark photoperiod. The fish were not fed during the 14 days of the experiment in order to avoid the intervention of this factor [33]. 

In each of the four experimental variants, the respiration of the fish was evaluated by determining the oxygen consumption (by the closed respiratory chamber method) and the frequency of respiratory movements at intervals of 24, 48, 72, 96, and 336 h from the initiation of the experiments [33]. After 14 days of setting up the experiments, the fish were sacrificed, and blood samples were taken from the tail vein to determine the number of figure elements and blood glucose values. The fish were euthanized by immersion in an overconcentrated anesthetic solution (benzocaine hydrochloride 250 mg L^−1^) for at least 10 min after stopping opercular movements, followed by decapitation [34].

Counting of figured elements was carried out under an Olympus microscope CX 31 using Thomma counting chambers after prior staining with neutral red and crystal violet [33]. Glucose determination was performed from blood drops with an Accutrend Plus GCT device. 

Values are given as arithmetic means ± standard error of the mean (SEM). The data were statistically analyzed using multiple comparison tests (LSD—SPSS/PC program version 10.0 for Windows). The statistical interpretation of the obtained results was carried out by means of the “one-way” ANOVA test for all investigated physiological parameters, comparing the results obtained within the groups exposed to Coragen 20 SC with those within the control group, after the same time intervals from the mounting of the experiments.

### 2.2. The Evaluation of Coragen-Induced Phytotoxicity

The phytotoxicity was carried out using the *Triticum* test, and the following parameters were determinate: growth in the length of the root and stem of the seedlings, wet and dry weight and photosynthetic pigments. 

The seeds of *Triticum aestivum* L. (Trivale variety) obtained from the Agricultural Research and Development Station, Albota (Argeș county, Romania) were sterilized (5 min in 75% ethanol) and then immersed in distilled water for 1 h. After immersion in the Coragen 20 SC solution (0.0125, 0.025, 0.05 mL L^−1^) for 1 h, the seeds were then placed on filter paper in Petri dishes and stored in a growth chamber (POL-EKO KK 350) at 25 °C during daytime and 15 °C at night, photoperiod: 16 h of light and 8 h of darkness. For the control, we used only distilled water. 

**Figure 1:**
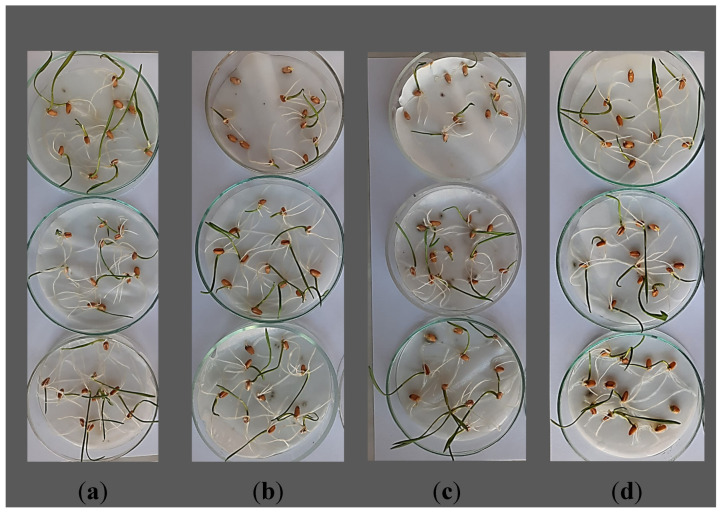
*Triticum aestivum* L. seedlings 7 days after exposure to Coragen 20 SC insecticide: (**a**) control, (**b**) 0.0125 mL L^−1^, (**c**) 0.025 mL L^−1^, (**d**) 0.05 mL L^−1^.

The root and stem length measurements were made 7 days after the exposure. The appearance of seedlings 7 days after the immersion in Coragen 20 SC can be seen in Figure 1a–d. After the fresh weight was determined, the plant material was placed in the oven at 80 °C to determine the dry weight. 

The evaluation of the physiological response of *Triticum aestivum* to Coragen 20 SC exposure was carried out by determining the content of photosynthetic pigments—chlorophyll *a* and *b* and carotenoids from an acetone extract—using a spectrophotometric method with Holm’s formulae [35]. The biological material was 30-day seedlings obtained after a similar protocol (immersion: 1 h in distilled water and 1 h in Coragen 20 SC solution). The absorbance of the samples was measured using a spectrophotometer (UV-VIS T70+). 

The inhibition rate of the length of the root and stem seedlings and of the photosynthetic pigment content was calculated using Formula (1) provided by Ma et al. [36]:

The inhibition rate = [(Gm − Gx)/Gm] × 100(1)

Gm—values reached for the control-determined parameters, Gx—values reached for the determined parameters for the variants exposed to Coragen 20SC. Ten seeds were used for each variant, and each experiment had 3 replications. 

The statistical interpretation of the data was made using IBM SPSS Statistics 20. The values are the means of 3 repetitions ± standard error; a, b—Duncan test results. Comparisons were made between control and Coragen 20SC variants (*p* = 0.05).

## 3. Results and Discussion

### 3.1. Influence of Coragen 20 SC on Perca Fluviatilis (Linné, 1758)

#### 3.1.1. Effects on Oxygen Consumption and Respiratory Rate

As a result of exposure to chemical pollutants, fish suffer physiological alterations (disruption of sensory, hormonal, neurological and metabolic systems) and behavioral changes [37]. The analysis of oxygen consumption can be used as a bio detector system to evaluate the toxic potentiality of the chemical [38]. Respiration rate and oxygen consumption in perches exposed to Coragen 20 SC insecticide at three concentrations (0.0125; 0.025 and 0.05 mL L^−1^) are shown in Table 1 and Table 2. 

Following exposure to the Coragen 20 SC insecticide at a concentration of 0.0125 mL L^−1^, no significant changes in the oxygen consumption of the perch occurred in the first 72 h. After 96 h of exposure, a slight significant increase in this parameter was observed (by 6.27% compared to the control group), the values recorded after 168 h and 336 h, respectively, being significantly lower than the control ones (by about 5%). In the case of the other two concentrations of Coragen 20 SC investigated, the effect was a decrease in oxygen consumption (significant decrease after 48 h, respectively 24 h), the decrease continuing until the 14th day (by 10.69%, respectively 15.55% comparative with the control lot).

The respiratory rate of the perch exposed to Coragen 20 SC in different concentrations had a similar evolution: an increase in this parameter in the first 96 h, 72 h, 24 h after contact with the insecticide, the period of stimulation of the frequency of respiratory movements being inversely proportional with the amount of insecticide.

The increase in respiratory rate in the first days of exposure can be attributed to the attempt to recover the oxygen deficit established as a result of exposure to insecticides. The respiratory rhythm values determined at the end of the experimental period were, in all experimental variants, lower compared to the control group (88.52%, 90.43% and 82.78%, respectively).

Venkata Rathnamma and Nagaraju (2014) [38] reported a decreased oxygen consumption rate in *Labeo rohita* specimens exposed to sublethal and lethal concentrations of CAP. The authors also reported severe respiratory distress, rapid opercular movements, increased mucus secretion, higher ventilation volume, labored breathing and engulfing of air through the mouth observed in the fish. An increase in opercular fish movement was initially observed, but it later decreased with an increased period of exposure to CAP [37,39]. 

In fish, pesticides first pass through the gills, so any disorder at this level will have a great influence on the adaptive changes. The oxygen uptake capacity of the gills is closely related to their histological integrity. The toxic effects of the environmental contaminant CAP on the gill of freshwater fish *L. rohita* exposed to sub lethal concentrations (1/10th 96 h of LC50) for 15 days include epithelial lifting, aneurysm, necrosis, and degeneration of secondary lamellae [38]. Gill damage is assumed to be responsible for respiratory distress. 

#### 3.1.2. Effects on Hematological Parameters, Glucose, and Behavioral Changes

The number of red blood cells and the blood glucose values signaled the state of stress in fish, which is why we recommended using these parameters as biomarkers of toxic stress induced by pesticides.

Although pesticides are hematological stressors in fish, there is no pattern of changes in these parameters for all classes of pesticides [40]. Monitoring hematological parameters in fish from pesticide-polluted waters can contribute to the early detection of this type of pollution [40,41,42].

Fish change their energy metabolism, spending a larger amount of energy to mitigate toxic stress [43]. Plasma glucose level is frequently used as a parameter to assess stress in aquatic organisms [44].

The mean number of red blood cells, white blood cells and plasma after exposure for 14 days to Coragen 20 SC in different concentrations is presented in Table 3.

No significant changes were recorded in the average number of erythrocytes and leukocytes in fish exposed for 14 days to Coragen 20 SC in a concentration of 0.0125 mL L^−1^. In the case of groups II and III (Coragen 20 SC 0.025 mL L^−1^ and 0.05 mL L^−1^, respectively), the average number of erythrocytes and leukocytes decreased significantly compared to the control group (by 12% and 17.9% in the case of erythrocytes and with 12.24% and 16.11%).

Significant changes in red blood cell count, white blood cell count and hematocrit values following exposure to Coragen have also been reported in homeotherms [45,46]. 

Decreased leukocyte levels may be suggestive of potential immunomodulation, phagocytic activity being a commonly used parameter to assess the immunotoxic potentials of chemicals [47]. The cellular components of the fish’s innate immune system consist of many different types of cells, such as monocytes/macrophages; granulocytes such as mast cells/eosinophilic granule cells, and neutrophils; dendritic cells; and natural killer cells (NK cells) [48]. The pesticides affected specific immune cells, causing apoptosis, changes in factor nuclear kappa B (NF-κB) expression, pro-inflammatory factors interleukin 6 (IL-6), interleukin 8 (IL-8), interferon-gamma (IFN-γ), chemokines (CXCL-c1c) and anti-inflammatory factor (interleukin 10 (IL-10) [49]. Blood glucose, the energy underlay of different reactions (although not as important as in mammals), significantly changed in the insecticide samples. Average blood glucose values were significantly higher after 14 days of exposure to Coragen 20 SC insecticide for all concentrations tested (by 9.1% higher in the case of perch exposed to 0.0125 mL L^−1^ insecticide compared to the values recorded in the control group, by 15.19% in those exposed to 0.025 mL L^−1^, and by 21.94% in those exposed to 0.5 mL L^−1^). 

Our observations agree with those of Nagaraju and Venkata Rathnamma (2018) [50], who reported a decrease in glycogen levels in the liver, muscles, gills and kidneys in *Channa punctatus* species exposed for 45 days to CAP at a concentration of 1442 mg L^−1^ (semistatic test with solution change at 24 h), a characteristic effect of fish exposed to pesticides; in this instance, the glycogen is broken down into glucose and used in accordance with the new energy needs of the intoxicated animals. Other works in the specialized literature indicate lethal values of over 10 mg L^−1^; the LC50 96 h for the insecticide CAP for the fresh water fish *Channa punctatus* was found to be 14.4 mg L^−1^ [51], 11.0 mg L^−1^ for the grass carp *Ctenopharyngodon idella* [39], 16.465 mg L^−1^ for the fish *Cirrhinus mrigala* [38], and 12.7 mg L^−1^ for *Labeo rohita* [39]. 

In acute static studies (96 h) performed on specimens of *Oncorhynchus mykiss*, *Lepomis macrochirus*, *Ictalarus punctatus*, and *Cyprinodon variegatus* exposed to CAP, no mortality or sublethal effects were reported at values up to 13.8, 15.1, 13.4 and 12 mg insecticide L^−1^, respectively [12,14]. 

Chen et al. (2022) [52], in a study on the safety of aquatic organisms in relation to the insecticide CAP, reported that the product was less toxic to *Brachydanio rerio*, with a 10.2 mg L^−1^ dose for 4-day LC50 acute toxicity. 

During the entire duration of the experiments (14 days), we did not find mortality in the groups studied. The LC50 96 h value of CAP (Coragen) on the *Channa punctatus* was found to be 12.7 mg L^−1^ [39], which agrees with our observations. 

However, we observed behavioral changes in the fish of groups II and III (Coragen 20 SC 0.025 mL L^−1^ and 0.5 mL L^−1^) right from the first 24 h after exposure to the insecticide: hyperactivity, jerky movements, muscle contractions, lack of movement coordination, seizures, ataxia hyper secretion of mucus. 

A similar behavioral pattern was observed during the exposure period to the insecticide CAP; jerky movements, hyper secretion of mucus, opening and closing of the mouth for gasping, loss of scales, and hyperactivity was observed in the experimental group [38,39]. They slowly became lethargic and restless, and they secreted excess mucus all over their body. 

### 3.2. Influence of Coragen 20 SC on Triticum aestivum Seedling Growth and Weight

**Figure 2:**
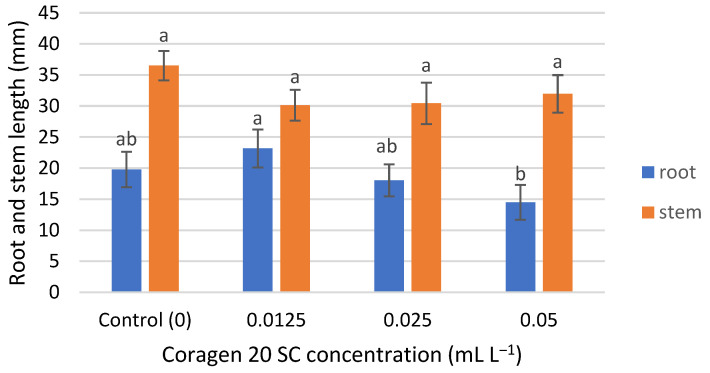
The influence of Coragen 20 SC on *Triticum aestivum* L. root and stem length after 7 days of exposure (a–b: Interpretation of the significance of differences using Duncan’s test, *p* = 0.05).

*T. aestivum* seedling root growth was insignificantly influenced by Coragen 20 SC, being slightly stimulated in the case of the lowest concentration (0.0125 mL L^−1^) and slightly inhibited at the higher applied concentrations (0.025 and 0.05 mL L^−1^) (Figure 2), with an inhibition rate of 8.75% and 26.61%, respectively. Stem seedling growth was inhibited at all concentrations tested, but the differences were not significant (Figure 2). The inhibition percentages were between 12.43% (for the 0.05 mL L^−1^ Coragen 20 SC experimental variant) and 17.45% (for the 0.0125 mL L^−1^ Coragen 20 SC experimental variant). This could be explained by the fact that the pesticide can translocate from the root to the stem and leaves, as reported in some research. According to Fan et al. (2021) [53], CAP could be absorbed by roots under hydroponic conditions and transported to the upper parts of the plant. Also, a bioconcentration of CAP was observed in maize roots, and absorption occurred via an apoplastic pathway. The cell wall was the dominant storage compartment. The root uptake rate constant (k1) for CAP is low (0.001 h^−1^) compared to other pesticides (difenoconazole) (0.699 h^−1^). Also, the shoot uptake rate constant (k2) of CAP was smaller than those of k1, and the transport from the root to the stem was a slow one [54]. Damage to aquatic plant growth was reported in *Lemna gibba* (Duckweed) at concentrations greater than 2 mg L^−1^, and likewise for reproduction at 2 mg L^−1^ [22]. 

**Figure 3:**
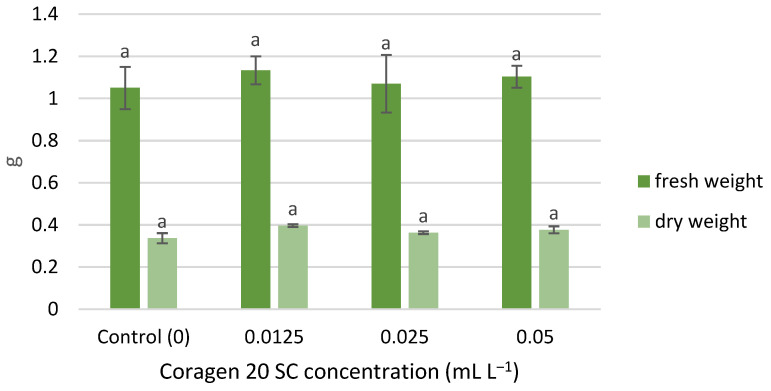
The influence of Coragen 20 SC on *Triticum aestivum* L. fresh and dry weight, after 7 days of exposure (a-: Interpretation of the significance of differences using Duncan’s test, *p* = 0.05).

The wet and dry weight of *T. aestivum* seedlings was slightly stimulated by exposure to Coragen 20 SC, following the same growth pattern, in the following order of variants: 0.0125, 0.05, 0.025 mL L^−1^ (Figure 3). However, the differences recorded between the samples were not significant. Similar results have been reported for peppermint. Thus, the treatment with CAP and other insecticides (imidacloprid, pyriproxyfen, acetamiprid, and chlorfenapyr) on mint did not induce significant changes among stem length and fresh weight [55]; insecticide treatments increased the concentration of phytohormones (jasmonic acid and abscisic acid) in stem and leaves and, generally, decreased the contents of most phenolic acids [51].

### 3.3. Influence of Coragen 20 SC on Photosynthetic Pigments

**Figure 4:**
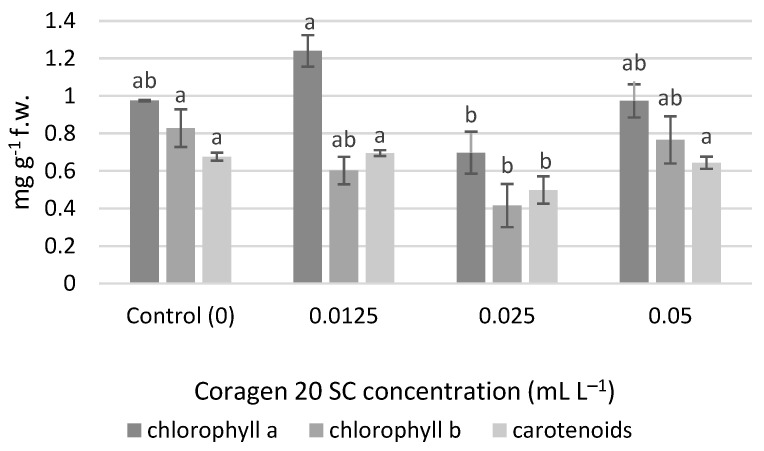
The influence of Coragen 20 SC on *Triticum aestivum* L. photosynthetic pigment content after 30 days of exposure (a–b: Interpretation of the significance of differences using Duncan’s test, *p* = 0.05).

No significant differences were determined for the chlorophyll content, but at a concentration of 0.0125 mL L^−1^ Coragen 20 SC, the amount of chlorophyll *a* (1.240 mg g^−1^ f.w.) was higher compared to the control (0.974 mg g^−1^ f.w.), and also compared to the variants 0.025 mL L^−1^ (0.697 mg g^−1^ f.w.) and 0.05 mL L^−1^ (0.973 mg g^−1^ f.w.) (Figure 4). An inhibition percentage of 28.43% was obtained in the variant 0.025 mL L^−1^ for chlorophyll *a*. The content of chlorophyll *b* and carotenoid pigments were significantly lower in the case of the variant exposed to 0.025 mL L^−1^ Coragen 20 SC. In the case of chlorophyll *b*, the inhibition percentages were 27.20% (0.0125 mL L^−1^), 49.81% (0.025 mL L^−1^) and 7.49% (0.05 mL L^−1^). In the case of carotenoid pigments, a slight stimulating effect was observed in the variant exposed to 0.0125 mL L^−1^ Coragen 20 SC (0.695 mg g^−1^ f.w.) compared to the control (0.676 mg g^−1^ f.w.), and the other two variants had recorded inhibition percentages of 26.33% (0.025 mL L^−1^) and 4.73% (0.05 mL L^−1^), respectively. 

Kilic et al. (2015) [56] reported significant changes in the amount of photosynthetic pigments in *Zea mays*, induced by CAP (0.08–0.5 ppm). These were accompanied by damage to the germination, the growth of the axial organs, the number of roots/seedlings, stomatal density and size. Other metabolic changes highlighted in other research were the increase in the amount of anthocyanins and proline, changes that constitute another response of plants to the stress represented by pesticides, including CAP [56,57]. In contrast, Lin et al. (2022) [55] did not reveal changes in chlorophyll pigments in mint leaves in the presence of CAP and imidacloprid, pyriproxyfen, acetamiprid, and chlorfenapyr. In the mentioned research, the authors obtained a reduction in the content of soluble proteins and an increase in the content of cinnamic acid and lignin in response to the stress induced by CAP and other pesticides.

The evaluation of non-enzymatic and enzymatic antioxidants in cultivated tomatoes, in which the efficiency of Coragen 20 SC against *Tuta absoluta* was evaluated, indicated an increase in the content of photosynthetic pigments; a decrease in the activity of the enzymes super oxide dismutase, catalase, polyphenol oxidase, glutathione-S-transferase; and a decrease in the amount of total phenols in the period between 1 and 7 days after performing the treatment in the field [20]. The authors explain the decrease in enzyme activity and the increase in the content of photosynthetic pigments as being because of the absence of abiotic stress, represented by insects. In agreement with other research [58,59], the authors established an inverse correlation between the content of photosynthetic pigments and antioxidant enzymes in the absence of insects.

The changes highlighted in the experiment carried out are explained by the complex action of pesticides, including carboxamides, on the synthesis of nucleic acids, mitosis, cellular respiration and plant defense induction, among the targeted action sites being cited: RNA polymerase I, DNA/RNA synthesis, β-tubulin assembly in mitosis, Complex II—succinate dehydrogenase Complex III—cytochrome bc1 (ubiquinol oxidase) Complex III—cytochrome bc1 (ubiquinone reductase), etc. [60].

In the presence of pesticides, plants activate the response pathways, which Parween and Jan (2019) [60] mention, respectively: detoxification processes by cytochrome P450 monooxygenases or glutathione-S-transferases (GSTs), induction of antioxidant enzymes, synthesis of phenolic compounds, and synthesis of pathogenesis-related (PR) proteins (PR-1 to PR-19) [61].

## 4. Conclusions

This paper is a contribution to the knowledge of toxicity and the effects of Coragen 20 SC insecticide on two non-target organism species, *Perca fluviatilis* (Linné, 1758) and *Triticum aestivum* L. 

The study of the oxygen consumption and the respiratory rate, in conjunction with determining the number of red blood cells, white blood cells and the blood glucose level, as well as the main behavioral changes, allowed us to form a table of the overall symptoms of the perch poisoned with CAP insecticide. The analyzed insecticide reduced the energy metabolism and the respiratory rate, decreased red blood cells and white blood cells, increased glucose level in the blood, and induced behavioral changes in the perch. 

The results obtained during the evaluation of Coragen 20 SC toxicity using the *Triticum* test indicate a weak to moderate phytotoxicity for the parameters considered and at the doses applied. Only the assimilatory pigments were significantly modified at the concentration of 0.025 mL L^−1^ for the growth of the axial organs and the wet and dry weight, the changes obtained not being statistically significant. 

Further testing is needed to highlight other sequences in the possible response pathways that plants and animal activate in the presence of the insecticide; further testing is also needed for evaluation with other terrestrial, aquatic, wild and/or crop species. 

## Figures and Tables

**Table 1:** Variations in the average oxygen consumption (mL oxygen/kilogram/hour) and the standard deviation of the *Perca fluviatilis* (Linné, 1758) exposed to Coragen 20 SC insecticide at different concentrations.

Lots	0 h	24 h	48 h	72 h	96 h	168 h	336 h
Control	265 ± 12.41	256 ± 11.36	262 ± 8.44	270 ± 9.62	258 ± 11.52	267 ± 9.45	261 ± 12.56
Lot I Coragen 20 SC 0.0125 mL L^−1^	256 ± 7.62	260.24 ± 8.42	262.25 ± 11.42	264.14 ± 7.66	274.2 ± 8.52 *	258.3 ± 14.83	248.23 ± 6.68 *
Lot II Coragen 20 SC 0.025 mL L^−1^	264.4 ± 8.14	258.4 ± 8.62	254.5 ± 9.58 *	246.42 ± 8.12 *	234.3 ± 8.98 *	238.46 ± 4.47 *	229.52 ± 7.78 *
Lot III Coragen 20 SC 0.05 mL L^−1^	272.7 ± 9.12	246.8 ± 7.14 *	238.5 ± 11.52 *	230.42 ± 8.62 *	224.3 ± 8.96 *	218.32 ± 8.79	220.42 ± 9.64 *

* The mean difference was significant at the 0.05 level.

**Table 2:** Variations in the average respiratory rate (breaths/minute) and the standard deviation of the *Perca fluviatilis* (Linné, 1758) exposed to Coragen 20 SC insecticide at different concentrations.

Lots	0 h	24 h	48 h	72 h	96 h	168 h	336 h
Control	73.2 ± 3.2	72.7 ± 3.22	74.94 ± 8.28	72.6 ± 4.74	71.5 ± 8.56	72.8 ± 5.42	73.2 ± 4.48
Lot I Coragen 20 SC 0.0125 mL L^−1^	72.6 ± 3.48	78.8 ± 2.38*	84.2 ± 3.42 *	82.4 ± 6.36 *	79.3 ± 4.28 *	68.3 ± 2.82	64.8 ± 2.42 *
Lot II Coragen 20 SC 0.025 mL L^−1^	78.2 ± 7.22	82.6 ± 4.12	86.3 ± 8.48 *	82.8 ± 4.18	75.8 ± 5.62	65.8 ± 5.28 *	66.2 ± 8.42 *
Lot III Coragen 20 SC 0.05 mL L^−1^	72.8 ± 5.54	79.5 ± 3.12	75.6 ± 4.42	67.8 ± 2.18 *	63.1 ± 4.24 *	61.2 ± 2.46 *	60.6 ± 5.42 *

* The mean difference was significant at the 0.05 level.

**Table 3:** The mean number of figured elements and glucose with standard deviation in *Perca fluviatilis* (Linné, 1758) exposed to Coragen 20 SC.

Lots	Red Blood Cells/mL Blood	White Blood Cells/mL Blood	Glucose (mg/100 mL Blood)
Control	1,316,415 ± 8380	53,950 ± 5820	88.76 ± 11.20
Lot I Coragen 20 SC 0.0125 mL L^−1^	1,302,460 ± 11,550	52,550 ± 4750	96.84 ± 5.42 *
Lot II Coragen 20 SC 0.025 mL L^−1^	1,158,450 ± 128,150 *	47,350 ± 6240 *	102.25 ± 6.85 *
Lot III Coragen 20 SC 0.05 mL L^−1^	1,080,840 ± 10,750 *	45,260 ± 5640 *	108.24 ± 9.12 *

* The mean difference is significant at the 0.05 level.

## Data Availability

Data are contained within the article.
